# Ankyloglossia severity in infants: maternal pain, self-efficacy, and functional aspects of breastfeeding

**DOI:** 10.1590/1984-0462/2024/42/2022203

**Published:** 2023-10-23

**Authors:** Christyann Lima Campos Batista, Alex Luiz Pozzobon Pereira

**Affiliations:** a Universidade Federal do Maranhão, São Luís, MA, Brazil.

**Keywords:** Ankyloglossia, Breast feeding, Self efficacy, Pain, Anquiloglossia, Aleitamento materno, Autoeficácia, Dor

## Abstract

**Objective:**

To analyze functional aspects of breastfeeding, self-efficacy, and pain reported by mothers during breastfeeding, in newborns with severe and mild ankyloglossia.

**Methods:**

This is an observational study, carried out with 81 babies with ankyloglossia, assessed by the Bristol Tongue Assessment Tool (severe: scores 0–3; mild: scores 4–6) nested in a cohort carried out at the University Hospital of the Federal University of Maranhão, São Luis, Brazil. The functional aspects of breastfeeding were analyzed using the Breastfeeding Observation Form of the United Nations Children's Fund (BOF-UNICEF) and the LATCH Scoring System. Breastfeeding self-efficacy was measured using the Breastfeeding Self-Efficacy Scale — Short-Form. Pain indicators were evaluated by the Short-Form McGill Pain Questionnaire. The significance level adopted was 5%.

**Results:**

Maternal age was 26.7±0.8 years, and 64.2% reported high school education. Most babies were male (67.9%), and the birth weight was 3232±60g. A significant association was detected in the sucking aspect evaluated by the BOF-UNICEF [β=0.22 (95%CI 0.07; 0.73), p-value=0.013]. However, the groups did not differ in the assessment of breastfeeding performed by the LATCH scale. The groups had no differences in the assessment of breastfeeding self-efficacy reported by mothers, and in pain scores.

**Conclusions:**

Despite the observation of sucking difficulty in infants with severe ankyloglossia., the quality of breastfeeding in general, maternal pain, and self-efficacy reported by mothers do not differ when compared with infants with mild ankyloglossia. Therefore, the severity of ankyloglossia seems not to affect the breastfeeding indicators.

## RESUMO

**Objetivo:**

Analisar aspectos funcionais da amamentação, autoeficácia e dor relatada pelas mães durante a amamentação, em recém-nascidos com anquiloglossia grave e leve.

**Métodos:**

Estudo observacional, realizado com 81 bebês diagnosticados com anquiloglossia avaliados pelo *Bristol Tongue Assessment Tool* (grave: escores 0–3; leve: escores: 4–6), aninhados em um estudo de coorte realizado no Hospital Universitário da Universidade Federal do Maranhão, São Luís, Brasil. Os aspectos funcionais do aleitamento materno foram analisados por meio do Formulário de Observação da Amamentação do Fundo Internacional para a Infância das Nações Unidas (BOF-UNICEF) e da Escala LATCH. Aautoeficácia foi mensurada por meio da *Breastfeeding Self-Efficacy Scale — Short-Form*. Os indicadores de dor foram avaliados pelo *Short-Form McGill Pain Questionnaire*. O nível de significância adotado foi de 5%.

**Resultados:**

A idade materna foi 26,7±0,8 anos, e 64,2% concluíram o ensino médio. A maioria dos recém-nascidos era do sexo masculino (67,9%), e o peso ao nascer foi 3232±60g. Foi detectada associação significante no aspecto da sucção avaliado pelo BOF-UNICEF [β=0,22 (IC95% 0,07; 0,73), p=0,013]. No entanto, os grupos não diferiram na avaliação do aleitamento materno realizada pela escala LATCH. Os grupos não apresentaram diferenças na avaliação da autoeficácia em amamentar relatada pelas mães e no escore de dor.

**Conclusões:**

Apesar de ter sido observada dificuldade de sucção em lactentes com anquiloglossia grave, a qualidade da amamentação em geral, a dor materna e a autoeficácia relatada pelas mães não diferem quando comparadas com as de lactentes com anquiloglossia leve. Assim, a gravidade da anquiloglossia parece não afetar os indicadores de amamentação.

## INTRODUCTION

The restriction of tongue movement due to ankyloglossia has been reported as a cause of significant functional loss in the sucking pattern and latch unattachment, in a sensitive period of the infant's development. Among the symptoms reported in the literature, reports of severe pain when breastfeeding, difficulties in maintaining the handle on the mother's breast, mechanical trauma such as cracks and nipple abrasions, gastrointestinal symptoms, and even low weight gain are common.^1^ The epidemiology varies according to the age of the individual, or the diagnostic method used, but the prevalence of the alteration can reach 8%,^2^ and symptomatic alterations were seen in up to 70% among babies diagnosed with tongue tie.^3^

The condition is characterized by the shortening or poor insertion of a band of submucosal connective tissue, a dynamic structure that connects the wall of the lower mandibular arch to the floor of the mouth, which is responsible for a balance between mobility and stability of lingual movement.^4^ Ankyloglossia is a hereditary condition; it is more prevalent in male babies, but not all cases can be explained by genetics.^2^ Clinical procedures or even surgical corrections may be necessary in some cases to improve indicators related to breastfeeding.^5^

Human milk is well known as the best food source for all babies. Thus, the practice of exclusive breastfeeding (EBF) until the 6^th^ month of life has been recommended by governmental organizations and associations of health professionals.^6,7^ The short and long-term benefits of breastfeeding to the mother-infant dyad are more present in low- and middle-income countries, where low rates of breastfeeding are associated with higher mortality.^8^

Therefore, the scientific literature has been exhaustively dedicated to clarifying possible changes that may somehow interfere with this important health strategy. The mechanisms of sucking and muscle mobility of sensitive structures involved in breastfeeding play an important role in the development of stomatognathic structures, which will play important roles in the child's development, such as speech and deglutition disorders.^9^

Randomized studies describe an improvement in the functional aspects of breastfeeding; however, they focus only on surgical interventions, leading to an improvement in pain-related symptoms in the short term.^10^ There are few studies on the different degrees of severity of ankyloglossia, and the role of these gradations in breastfeeding indicators such as pain and self-efficacy. The present study aims to clarify whether the degree of severity of ankyloglossia in healthy full term newborn infants may present a risk to the functional aspects of breastfeeding after the first week of life.

## METHOD

A cross-sectional observational study was conducted, nested within the cohort “Can ankyloglossia neonatal interfere with the prevalence of breastfeeding and the evolution of the growth of babies during the first six months of life? A cohort study”. Data from 81 newborns and their mothers were evaluated, this being a sub-sample of the cohort that took place at the University Hospital of the Federal University of Maranhão, Maternal-Infant Unit (HUUFMA), in the sectors Human Milk Bank (HMB) and rooming-in.

The data collection period was between July 2019 and July 2021. The HUUFMA is a high-complexity hospital, specialized in obstetric and neonatal care for high-risk mother-child dyads in the state of Maranhão, located in the city of São Luís, with an average of 330 deliveries/month. In Brazil, HMB is also a reference center for difficulties in breastfeeding in the public health system.

This study was approved by the Research Ethics Committee of the institution (report #3.052.208). All mothers were informed about the risks and benefits of the study, and they all agreed to participate by signing the Free and Informed Consent Form. At all stages of the study, compliance with current national legislation dealing with conducting research with human beings was maintained.

Term infants born without problems that could compromise breastfeeding or development (cardiac, neurological, pneumological diseases or congenital syndromes) were included in this study in the rooming-in sector before hospital discharge. Babies from multiple pregnancies and preterm were not included. The sample was non-probabilistic, and data were collected from patients who attended the follow-up consultation around 28 to 37 days after delivery.

Ankyloglossia diagnoses were performed using the Bristol Tongue Assessment Tool — BTAT,^11^ a validated instrument witha simple application for screening. It has two items for assessing appearance, and two items for functional assessment, with a result between 0 and 8.

Infants with scores lower than, or equal to 6, were included in the study. They were classified into two groups: infants with severe ankyloglossia (with test results between 0 and 3), and infants with mild cases (with test results between 4 and 6). This classification followed the determination of the original authors of the test. Assessments were performed by two speech therapists trained to use the diagnostic test; they participated in a 20-hour theoretical and practical guidance class. Each infant was evaluated by both professionals in a different moment (at rooming-in, while the infant was in the hospital). This assessment is part the institution's neonatal screening program.

Those infants with results indicative of alterations in the lingual frenulum were then referred for follow-up at the HMB of HUUFMA, where aspects related to breastfeeding (observation of the health professional and maternal self-assessment), and pain during breastfeeding were evaluated.

Information on obstetric and neonatal data was collected from discharge summaries and used to characterize the sample. The infants and their mothers then proceeded to clinical assessments of breastfeeding, maternal self-perception of breastfeeding efficacy, and their impression of breastfeeding pain at the follow-up consultation (maximum 37 days after delivery).

Two instruments were used to assess breastfeeding. The first was the LATCH Scoring System scale,^12^ an easy-to-apply instrument that assesses five aspects of breastfeeding (latch, audible swallowing, type of nipple, comfort and hold), with a maximum score of 10. Scores less than, or equal to 8, were considered indicative of difficulties in breastfeeding.

The second instrument was the Breastfeeding Observation Protocol of the United Nations Children's Fund and the World Health Organization — BOF-UNICEF,^13^ which details breastfeeding aspects in five domains: position, responses, establishment of affective bonds, anatomy, and sucking. To classify breastfeeding difficulties, a methodology was used to classify negative behaviors into good, regular, and bad, as shown in Table 1.

**Table 1:** Criteria for classifying the quality of the aspects evaluated in the Breastfeeding Observation Form (BOF-UNICEF).

Evaluated aspects	Number of negative behaviors investigated	Observed negative behaviors and classification		
		Good	Regular	Bad
Position	05	0–1	2–3	4–5
Responses	06	0–1	2–3	4–6
Sucking	06	0–1	2–3	4–6
Anatomy	04	0	1	2–4
Emotional Bonding	03	0	1	2–3

**Table 2:** Descriptive characteristics of the sample, newborn/maternal data and obstetric information of the participants evaluated in the study, São Luís, Brazil, 2022.

Variables			n	%
Newborn data				
	Baby sex			
		Male	55	67.9
		Female	26	32.1
	Apgar score 1^st^ minute (median; P25–P75)		9	(8–9)
	Apgar score 5^th^ minute (median; P25–P75)		9	(8–9)
	Skin-to-skin contact		69	85.2
	Breastfed at 1^st^ hour		44	54.3
Prior breastfeeding problems				
		Yes	32	40.5
		No	47	59.5
		Missing	2	2.5
Maternal data				
		Maternal age (mean; SD)	26.74	0.76
Marital status				
		With partner	60	74.1
		Single/Widow/No partner	21	25.9
Maternal education				
		Elementary or less	14	17.3
		High school	52	64.2
		Above high school	15	18.5
Occupation				
		Does not work	37	45.7
		Have a job	44	54.3
Family income				
		A minimum wage or less	13	16
		Between one and three minimum wages	21	25.9
		Between three and five minimum wages	44	54.3
		Above 5 minimum wages	3	3.7
Obstetric and prenatal data				
Type of delivery				
		Vaginal	43	53.1
		C-section	38	46.9
		Gestational age (median; PV)	39	38-40
Number of mother's births				
		1	48	59.3
		2	20	24.7
		3 or more	13	16
Prenatal				
		At HUUFMA	25	30.9
		Other public maternity hospitals	53	65.4
		Other private maternity hospitals	3	3.7

P25–75: 25 and 75% percentiles; SD: standard deviation; HUUFMA (University Hospital of the Universidade Federal do Maranhão).

Maternal confidence in breastfeeding was measured with the Breastfeeding Self-Efficacy scale (BSES/SF) completed by the mother herself.^14^ It is a Likert scale containing 14 statements in which it is possible to validate responses from 1 (totally disagree) to 5 (totally agree). The total score is 70 points; the higher the score, the greater the mother's confidence in the potential to breastfeed, thus showing a higher probability of maintaining breastfeeding.

Nursing mothers rated pain perception using the Short Form scale of the McGill pain Questionnaire — SF/MQP,^15^ comprising three sessions:

- 15 definitions of types of pain, which can be classified from 0 (none) to 3 (severe), maximum score of 45 points;
- visual analogue scale with scores from 0 (no pain) to 10 (maximum pain possible), and
- description of recent pain with descriptors, totaling 5 points.

The scale allows a total of 60 points; the higher the score, the greater the maternal perception of pain related to breastfeeding.

IBM's Statistical Package for the Social Sciences (SPSS) (version 26) was used. Data were collected in clinical protocols and then stored in a database for analysis. Numerical variables were tested for their distribution and described according to the Shapiro-Wilk test result.

Categorical variables were described in frequencies and percentages. To analyze the association between the exposure variable (ankyloglossia) and categorical outcomes, the chi-square (χ²) or Fisher's exact test was used, as indicated in the tables. To analyze the difference between the medians of the study groups, the Mann-Whitney U test was used.

Regression models were performed using the severity of ankyloglossia (0 mild, 1 severe), multinomial logistic regression for categorical scale, and a general linear model for scale variables. p<0.05 was considered statistically significant with a confidence interval of 95% (95%CI).

## RESULTS

The sample evaluated in this study showed that the babies followed were mostly male (67.9%), born with good vitality and had good delivery and birth practices such as skin-to-skin contact, and breastfeeding in the first hour. The most frequent maternal educational level was high school (64.2%), the most frequent marital status was “with a partner” (74.1%), mothers had some type of occupation (54.3%), and had a mean age of 26.7years (standard deviation — SD=0.7).

Most babies with ankyloglossia were vaginal born (53.1%), had a median gestational age of 39 weeks at birth, were the first child of the nursing mother, and underwent prenatal care in public health units. The descriptive data of the studied sample are described in Table 1.

The birth data of the babies evaluated in the study (birth weight, head circumference, thoracic perimeter, and length) has no significant association between the two groups of ankyloglossia severity. The data is presented on Table 3.

**Table 3:** Association between birth measures in infants with severe *versus* mild ankyloglossia, São Luís, Brasil, 2022.

	Total	Ankyloglossia		p-value*
		Severe	Mild	
	Mean (SD)	Mean (SD)	Mean (SD)	
Birth weight (g)	3232 (60)	3198 (125)	3241(69)	0.772
Head circumference (cm)	34.5 (0.2)	34.1 (0.4)	34.5 (0.2)	0.391
Thoracic perimeter (cm)	33.0 (0.3)	33.2 (0.7)	33 (0.3)	0.765
Length (cm)	48.6 (0.3)	47.9 (0.4)	48.8 (0.3)	0.209

* Student's t-test. SD: standard deviation; g: grams; cm: centimeters.

In the aspects evaluated in the BOF-UNICEF, presented in Table 4, only the sucking aspect showed a significant association with the severity of ankyloglossia (p=0.034). It was observed that 64.7% of the babies with severe tongue-tie presented a sucking behavior considered regular; they also presented high frequency of behaviors that were considered appropriate.

**Table 4:** Association between aspects of the Breastfeed Observation Form (BOF-UNICEF) and the severity of ankyloglossia, São Luís, Brazil, 2022.

	Good		Regular		Bad		p-value
	Severe	Mild	Severe	Mild	Severe	Mild	
Position	9 (52.9)	37 (57.7)	5 (29.4)	20 (31.7)	3 (17.6)	6 (9.5)	0.642*
Answers	11 (64.7)	54 (85.7)	5 (29.4)	6 (9.5)	1 (5.9)	3 (4.8)	0.071†
Affective Ties	9 (52.9)	26 (41.3)	4 (23.5)	16 (25.4)	4 (23.5)	21 (33.3)	0.693*
Anatomy	12 (70.6)	49 (77.8)	5 (29.4)	12 (19)	0 (0)	2 (3.2)	0.695†
Sucking	5 (29.4)	39 (61.9)	11 (64.7)	19 (30.2)	1 (5.9)	5 (7.9)	0.034†

* chi-square test;

† Fisher's exact test. One sample unit did not present data from the Breastfeed Observation Form.

However, other instruments did not demonstrate that the severity of ankyloglossia was associated with functional impairments in the assessment of breastfeeding. It was observed that there was no association between breastfeeding assessment on the LATCH scale and maternal self-efficacy (p>0.05), as shown in Table 5. Similarly, despite the median pain score being higher in babies with severe ankyloglossia, this difference was not statistically significant when compared to less severe cases.

**Table 5:** Association between functional indicators of breastfeeding and pain with ankyloglossia severity, São Luís, Brazil, 2022.

		Total	Severe ankyloglossia		p-value
			Yes	No	
LATCH scores					
	Indicative of difficulties in BF	42 (54.5)	12 (75.0)	30 (49.2)	0.091*
	BF without indication of difficulties	35 (45.5)	4 (25.0)	31 (50.8)	
BSES-SF		59 (55–63)	62. (55–67)	59 (55–63)	0.177†
SF-MPQ		8 (4–14)	10 (5–15)	6 (4–12)	0.471†

* chi-square test;

† Mann-Whitney U test.

BF: breastfeeding; AM: breastfeeding maternal; BSES-SF: Breastfeeding Self-Efficacy Scale-Short Form; SF-MPQ: Short Form of the McGill Pain Questionnaire.

In the regression models, the severity of ankyloglossia tended to vary for sucking behavior; thus, the more severe the ankyloglossia, the greater the probability of the infant presenting regular-type changes in sucking [β=0.22 (95%CI 0.07; 0.73), p-value 0.013]. However, this model explained only approximately 5% of the sample. The other outcome variables were not related to the severity of ankyloglossia. The values are shown in Table 6.

**Table 6:** Regression models between outcome variables and exposure to ankyloglossia severity, São Luís, Brazil, 2022.

Variable		β (95%CI)	p-value	R*
BOF-UNICEF Sucking†				
	Good	ref	-	
	Regular	0.22 (0.67–0.73)	0.013	0.047
	Bad	0.64 (0.06–6.65)	0.710	
LATCH Score†		-0.11 (-1.06–8.49)	0.331	0.013
BSES-SF†		0.15 (-1.38–7.15)	0.182	0.023
SF-MPQ†		0.00 (-4.29–4.43)	0.975	0.000

* general linear model;

† multnomial logistic regression.

B: regression coefficient; CI: confidence interval; BOF-UNICEF: Breastfeeding Observation Form (only aspect suction); BSES-SF: Breastfeeding Self-Efficacy Scale-Short Form; SF-MPQ: Short Form of the McGill Pain Questionnaire.

The participants of this study do not perform surgical procedures to correct the ankyloglossia until the assessment at the follow-up consultation (near one month after delivery). Exclusive breastfeeding was reported by 73 mothers (90.1%), pacifier use was verified in three (3.7%), and baby bottles by ten (12.3%). In average, the infant had a weight gain of 1075 g in the first month, representing an average of 31.6 grams per day.

## DISCUSSION

This research analyzed indicators of breastfeeding in babies with ankyloglossia according to the severity of the alteration. Results showed that those with severe ankyloglossia did not show changes in breastfeeding assessments and pain score despite an aspect of breastfeeding, sucking, which has been shown to be associated with severe ankyloglossia. The data collected in this study may indicate that the changes in breastfeeding caused by tongue-tie may resemble other changes in the beginning of breastfeeding and can be overcome with clinical control measures.

The sucking difficulties found in this study were also reported with a similar sample in studies that evaluated the impact of ankyloglossia on breastfeeding. Campanha et al.^16^ reported that the probability of sucking difficulties was 36 times higher in babies with the disorder, compared to those without tongue-tie, using the United Nations International Children's Emergency Fund (UNICEF) protocol. In addition, high prevalence of sucking problems in babies with ankyloglossia was reported by Ferrés-Amat et al.^17^ Changes in the sucking pattern are common at the beginning of breastfeeding, and they can be aggravated by the limitation of the natural movement of the tongue, which plays an important role in the growth of stomatognathic structures and functions.^18,19^

However, the isolated change in sucking showed no influence on the general performance of breastfeeding. Although the scores in babies with severe alterations were higher, no significant association was found in the evaluation by the LATCH scale when compared to babies with mild ankyloglossia. Souza-Oliveira et al.^20^stated that the success of breastfeeding may be dependent on other conditions such as full-term delivery, guidelines on breastfeeding, and family income; emphasizing the need to assess breastfeeding before deciding on frenotomy/frenectomy as they did not find an association between ankyloglossia and breastfeeding.

In the literature, the statement that the condition is associated with changes in breastfeeding is recurrent^21,22^ Studies consequently focus on reporting the beneficial effect of surgical correction on breastfeeding indicators.^23^ The effects of changes in breastfeeding can be variable, while the assessments carried out in most studies are in the first weeks of life, a period in which breastfeeding difficulties are common. There is therefore a physiological bias in the diagnosis and observed outcome.

The findings of the present study did not reveal significant differences in the self-efficacy of breastfeeding between the groups studied; thus, severe ankyloglossia does not seem to interfere in this aspect in the mothers’ opinions. There are few reports available regarding the self-efficacy of breastfeeding in babies with this condition. The studies available in the literature have shown that surgical correction improves efficacy indicators, being important markers for the success of breastfeeding-related outcomes.^24–27^ The BSES-SF values reported in the literature were lower than those found in this study, approaching the values presented by the babies after one month of the procedure performed.

The explanation of this finding may lie in the fact that the babies who sought surgery were the ones who commonly had breastfeeding restrictions, such as difficulty in maintaining a good latch, signs of reflux, and painful breastfeeding with signs of nipple trauma caused by the alteration. In the present study, however, the babies were evaluated at the time of routine neonatal screening in a unit that constantly practices policies to encourage breastfeeding by health professionals. This might have helped to overcome the initial difficulties related to breastfeeding. Diercks et al.^28^stated that most patients with ankyloglossia can benefit from non-surgical interventions based on accurate breastfeeding assessment strategies.

The babies with severe alterations evaluated in this research did not show a significant difference in the pain score evaluated when compared to symptomatic infants with mild alterations. One placebo-controlled clinical trial showed a mean SF-MPQ score of 16.77 (SD=1.88) with a significant reduction to 4.9 (SD=1.46) after the surgical procedure.^29^ Pain has been one of the main findings reported in studies involving problematic feeding in babies with a present diagnosis.^30^ It is necessary to emphasize that clinical measures can result in improved latch, thus reducing the pain perceived by the mother when breastfeeding, but the literature indicates that for severe symptomatic cases, frenotomy, regardless of the technique used, is the safe procedure to be used.^5^

It is noteworthy that pain can be an impediment to the continuity of breastfeeding, it is therefore a relevant clinical finding for monitoring severe symptomatic cases, allowing early intervention, and preventing weaning.

The present study has limitations. The first one is regarding its design, as it is not possible to make an association between exposure and outcome. In addition, some mothers’ characteristics were not measured, such as maternal pain and breast anatomy, which could lead to a breastfeeding difficulty in general. The infants were assessed in the first four weeks postpartum, a period when breastfeeding difficulties are common. However, the outcomes found here are important for highlighting the effectiveness of government breastfeeding policies, and the evaluation of a team specialized in difficulties related to breastfeeding. These initiatives can improve breastfeeding and reduce the number of unnecessary procedures, as the functionality indicators evaluated in this study do not show significant differences compared to infants with mild alterations.

In conclusion, this study reveals that there is an association between the severity of ankyloglossia and the infant's sucking aspect. However, no significant changes were observed in the functional aspects of breastfeeding, self-efficacy, and pain reported by mothers when compared to infants with mild alterations in this sample of infants. New studies on this topic may clarify this relationship, by taking into account confounding factors in the puerperal period, thus better clarifying the influence of ankyloglossia on the quality of breastfeeding.

## Data Availability

The database that originated the article is available with the corresponding author.
